# Linear and non-linear relationships between job demands-resources and psychological and physical symptoms of service sector employees. When is the midpoint a good choice?

**DOI:** 10.3389/fpsyg.2022.950908

**Published:** 2022-09-29

**Authors:** Francisco J. Sanclemente, Nuria Gamero, Alicia Arenas, Francisco J. Medina

**Affiliations:** Department of Social Psychology, University of Seville, Seville, Spain

**Keywords:** linear and non-linear relationships, job demands, job resources, psychological and physical symptoms, service sector

## Abstract

Related to the research of working conditions, the link between organizational factors and health was traditionally analyzed using linear models. However, the literature analysis suggests inconsistencies in linear models predicting workers’ health levels. To clarify this issue, this exploratory research compares the linear and non-linear relationships between job demands-resources (task complexity, time pressure, contact with users, and job autonomy), and the psychological and physical symptoms of employees working in the main five service subsectors: commerce, horeca (hotels, restaurants, and cafés), public administration, education, and healthcare. With a final sample of 4,047 participants, our study data were extracted from the II Andalusian Working Conditions Survey. Following the theoretical framework of JD-R Model and considering the Vitamin Model theoretical approach for non-linear relationships, our results showed that there were significant differences among the five subsectors analyzed regarding the linear and non-linear relationships between job demands-resources and psychological and physical symptoms of employees. Furthermore, task complexity generated non-linear relationships in higher proportion than time pressure and contact with users. Likewise, non-linear relationships found showed a U-shape. Moreover, the findings of non-linear relationships suggested that medium levels of task complexity should not be exceeded to avoid further negative impact on psychological and physical symptoms for service sector employees, preserving their health. Finally, some general practical implications of work environment interventions are suggested.

## Introduction

Stressful working conditions and their negative impact on employees’ health are considered an important issue for both the scientific community and society, generating changes in policies and human resources management. In article 151 of the Treaty on the Functioning of the European Union, the EU recognizes how important improvements in working conditions are. Furthermore, European social partners recognized the relevance of psychosocial risks by signing the Framework Agreements on Work-related Stress in 2004 (EU-OSHA, 2004). In Europe, 25% of employees experience work-related stress, reporting that work affects their health negatively (Eurofound, 2016). Additionally, differences in working conditions between groups of employees are sector-related (Eurofound and EU-OSHA, 2014). These statements mean that the health of millions of employees is negatively affected by work stress in the EU and reveal differences among work activities. Likewise, the sixth European Working Conditions Survey (6th EWCS) indicates that Spanish employees are exposed to high work intensity, which is linked with a negative impact on their health and wellbeing, revealing a high proportion of job activities related to high levels of time pressure, task complexity, and contact with users or clients (Eurofound, 2016). Additionally, the Spanish labor market is characterized by a working population primarily occupied in the service sector (Instituto Nacional de Estadística [INE], 2021).

Guided by the magnitude of the negative impact of stressful working conditions on employees’ health, the purpose of this exploratory study was to investigate how employees in five main service subsectors are negatively affected in terms of psychosomatic symptoms due to specific job demands that act as work stressors. To contribute to this understanding, first, we separately analyzed psychological and physical symptoms. Second, job demands were considered as specific job demands (task complexity, time pressure, and contact with users). And third, the impact of these specific job demands and job autonomy as job resource on employees’ psychosomatic symptoms was analyzed in five main service subsectors with different job activities through linear and non-linear models. The service subsectors considered in our study were commerce, horeca (hotels, restaurants, and cafés), public administration, education, and healthcare. Our objectives were threefold. First, to examine what specific job demands-resources affect psychological and physical symptoms. Second, to analyze whether specific job demands-resources impacted the psychosomatic symptoms of employees in a linear or non-linear way, and to define the shape of these relationships. And finally, to determine whether specific job demands-resources had different effects on the five different subsectors analyzed. Therefore, the main contribution of this exploratory study was to provide a comprehensive overview of the effects of the three main job demands and job autonomy as job resource on the psychosomatic symptoms of employees in the service sector, considering the physical and psychological symptoms separately, and showing the linear and non-linear nature of these relationships detailed by subsectors. Specifically, considering the five main service subsectors of the service sector, our study will show how task complexity, time pressure and contact with users, as job demands, will have a linear versus non-linear impact on health of employees depending on the subsector. Moreover, our results will shed light on how job autonomy, as job resource, will show a linear versus non-linear pattern in different subsectors. Likewise, our study will show the differential effects of sociodemographic variables, such as gender, age, and job tenure, on the job demands-health relationship.

### Relationship between job demands and psychosomatic symptoms of employees

The negative effect of job demands on physical and psychological employees’ health has been shown in numerous studies (Karasek, 1979; Bakker et al., 2003; Väänänen et al., 2003; Bakker and Demerouti, 2007, 2013). Theoretical models that explain this impact, such as the Job Demands-Resources (JD-R) Model (Bakker and Demerouti, 2007) and the Vitamin Model (Warr, 1987) signal how effects of job characteristics are. The JD-R model is a comprehensive model characterized by differentiating organizational factors such as job demands that affect employees’ health and job resources as job autonomy that protects employees’ health (Bakker and Demerouti, 2007). Job demands are defined as physical, psychological, organizational, or social aspects of work that involve sustained effort and that have a physical and psychic toll (Bakker and Demerouti, 2013) signaling that job demands have effects on physical and psychological employees’ health. Likewise, job resources are those physical, psychological, organizational, or social aspects of work that can reduce job demands and associated physiological and psychological costs, achieving job goals, or stimulating personal growth, learning, and development (Bakker and Demerouti, 2013). Thus, job demands act as the main predictors of variables such as exhaustion or psychosomatic health problems, and job resources act as the main predictors of job satisfaction, motivation, and engagement. At the same time, there is an interaction between job demands and job resources so that the job resources decrease the impact of job demands on strain and, on the other hand, job demands increase the impact of job resources on motivation-engagement (Bakker and Demerouti, 2013). Although the JD-R model signals relationships between job demands-resources and their outcomes, the authors do not indicate if these relationships are linear or non-linear. However, the traditional research, based in the framework of JD-R and other models, assumed linear effect of job characteristics (Ferris et al., 2006). One of the most commonly statistical method used in traditional organizational research is the linear regression model. The linear regression model assesses whether one or more predictor variables explain the dependent variable, and is based in four main assumptions as linearity, homoscedasticity, independence, and normality. In this sense, Ferris et al. (2006) suggest that theoretical development and data analytic methodology created together a linear organizational science based in linear relationships.

However, the assumption of linearity implies ignoring other possible effects (Warr, 2007), leading in possible inconsistency in results when data are analyzed exclusively from a linear perspective (Ferris et al., 2006). Likewise, traditional research assuming linear relationships only is too simplistic, thus future research should focus on the dynamic relations among the concepts in the framework of JD-R model (Schaufeli and Taris, 2014). In this line, the Vitamin Model considers the effects of job characteristics on health and wellbeing as an analogy to the role of vitamins on physiological health. In consequence, further vitamin intake produces constant effects without an improvement in health, this constant effect occurs with vitamins C and E (CE pattern). In the worst of the cases this further intake could cause a decrease in health. This additional decrease effect occurs with vitamins A and D (AD pattern with an inverted U-shape), this way, job demands are identified as a job characteristic with an AD pattern effect on affective wellbeing with an inverted U-shape. This model assumes that both CE and AD effects are non-linear (Warr, 1987). Thus, the Vitamin Model (Warr, 1987) gives theoretical support to non-linear relationships between work conditions, health, and wellbeing. However, previous research on non-linear relationships between job conditions and personal outcomes shown in Table 1 suggest some contradictory findings. For example, Lawson et al. (2009) found only linear relationships between psychosocial working conditions and psychological health in a sample of state-based police officers. Nevertheless, Noblet et al. (2009) found curvilinear links between workload and psychological health in a similar sample.

**TABLE 1:** Summary of revised works about non-linear research by kind of sample, measure of job demands, relationships found, and variables involved.

Non-linear research papers	Sample			Job demands		Relationships		Variables
Authors/Year/Journal	*N*	Homo JA	HeteroJA	As one variable	Specific	Linear	Non-linear	
Binnewies and Wörnlein (2011) *Journal of Organizational Behavior*	112	X			X	X	X	Time pressure-creativity-job control-affect
Chung-Yan and Butler (2011) *Canadian Journal of Behavioural Science*	259		X		X		X	Job complexity-demands-abilities fit-job satisfaction- proactive personality
Debusscher et al. (2014) *PLoS ONE*	45–52 130	X			X	X	X	Task complexity-work pressure-task performance-state neuroticism
De Dreu (2006) *Journal of Management*	109 212		X		X	X	X	Workload-task conflict-relational conflict-innovation-resource scarcity-information exchange-collaborative problem solving-task interdependence-goal attainment
Lawson et al. (2009) *Health Promotion International*	587	X		X		X		Job demands-job control-social support-organizational justice-psychological health-job satisfaction
Maltarich et al. (2010) *Journal of Applied Psychology*	11,892		X	X		X	X	Cognitive job demands-cognitive ability-voluntary turnover-job satisfaction
Noblet et al. (2009) *Stress and Health*	2,085	X		X		X	X	Job demands-social support-psychological health-job satisfaction-organizational affective commitment
Parkes (2016) *Applied Ergonomics*	901	X		X		X	X	Job demands-job control- physical work environment-sleep-age
Reis et al. (2017) *European Journal of Work and Organizational Psychology*	54	X			X	X	X	Time pressure-job control-vigor-absorption
Rydstedt et al. (2006) *Work and Stress*	4,154 6,000	X		X		X	X	Job demands- job control-social support-job satisfaction-psychological health
Sawang (2012) *International Journal of Manpower*	307	X		X			X	Job demands-work engagement-social support
Schmitt et al. (2015) *Journal of Personnel Psychology*	191		X		X		X	Time pressure-work engagement-illegitimate task
Sheng et al. (2019) *International Journal of Stress Management*	67		X		X		X	Time pressure-work engagement-psychological capital-sleep quality
Tanskanen et al. (2016) *Journal of Happiness Studies*	7,867		X	X		X	X	Job demands-job control-social support-work engagement
Van Ruysseveldt and Van Dijke (2011) *Journal of Vocational Behavior*	11,099 9,738		X		X		X	Workload-workplace learning opportunities- autonomy
**Total number of papers**		**8**	**7**	**7**	**8**	**10**	**14**	

Homo JA, homogeneous job activity sample; Hetero JA, heterogeneous job activity sample; sample size: 8 papers with N < 500 and 7 papers with N > 500.

Additionally, Warr (1994) carried out several recommendations for detection of non-linear relationships. First, using large samples, more than 500 participants. Second, using homogeneous work activities and a wide range of work characteristics. Third, using specific (better than general) job demands. And finally, distinguishing between psychological health (mental health) and physical health. However, as we can see in Table 1, few studies have large numbers of participants, some of them analyzed heterogeneous job activities and some of them only considered psychological health. According to Warr’s suggestions, to analyze non-linear relationships, a large sample and a wide range of job characteristics are required. In this sense, the use of a large work conditions survey is a chance to test this research topic.

### The impact of job demands on the service sector

The service sector is composed of different subsectors with specific characteristics and job activities (Eurofound, 2014b). Regarding the health of service sector employees in Europe, it is emphasized that service-oriented sectors and workers employed in manual occupations experienced significantly worse working conditions in terms of exposure to physical and psychosocial risks (Eurofound, 2014a). Specifically, administrative services and food and beverage services showed lower job quality indicators such as low pay rates, irregular working time schedules, no control over working time, and high levels of exposure to physical and psychosocial risks (Eurofound, 2014a). Additionally, the Spanish labor market is characterized by a population mainly occupied in the service sector. According to the National Institute of Statistics (INE) in 2019 (before the COVID-19 pandemic situation) the percentage of people employed in the service sector in Spain was 75.5%. Likewise, data from the 6th European Working Conditions Survey EWCS (Eurofound, 2017) indicates the percentage of Spanish employees whose job involves working at very high speed (64%), working to tight deadlines (64%), being in situations that are emotionally disturbing (53%), handling angry customers (53%), dealing directly with people such as customers, pupils, patients (67%) and being required to hide their feelings (54%).

These data reveal that Spanish employees are exposed to high levels of time pressure, task complexity, and contact with users (Eurofound, 2017). Time pressure is defined as the extent to which employees feel they do not have enough time to finish their work or need to work faster than usual (Binnewies and Wörnlein, 2011), or the amount of work a person has, combined with the required speed to fulfill it (Debusscher et al., 2014). Task complexity can be defined from an objective or a subjective point of view. The objective perspective considers task characteristics independent of the task performer from a structuralist or resource requirements points of view. However, the subjective perspective considers task properties and task performer characteristics in an interaction viewpoint. Thus, task complexity is defined by Liu and Li (2012) from an objective perspective as the aggregation of intrinsic task characteristics influencing the performance of a task. In this sense, Campbell (1988) also defined task complexity as ‘related directly to the task attributes that increase information load, diversity, or rate of change’ (p. 43). However, from a subjective point of view, when the complexity of the task exceeds the capacity of the worker, the worker will perceive it as perceived psychological complexity (Liu and Li, 2012). In this line, task complexity is defined as an individual’s perception of how complex a task is (Debusscher et al., 2014). Finally, contact with users is a common characteristic of the job among the service sector referred to interactions with users or customers (Szczygieł and Bazińska, 2013).

The 6th EWCS also analyzed work intensity as an index related to the level of job demands, such as workload, job absorption in terms of mental and physical energy, jobs that require juggling with various demands, and difficulties performing tasks in the most effective way (Eurofound, 2016). Our analysis, based on the work intensity index of the 6th EWCS (see Table 2), indicates that the five service subsectors considered had different levels of time pressure and task complexity. Although the time pressure work intensity index is quite similar to our study variable, the task complexity work intensity index is mainly related to emotional job demands, while in our study, task complexity is related to high levels of attention, complicated or difficult tasks, computer jobs and intellectually and emotionally demanding jobs.

**TABLE 2:** Work intensity index by sector EU28 (%) and their correspondence with our study variables time pressure and task complexity.

Work intensity variables	Commerce and horeca	Public administration	Education	Healthcare
Working at high speed (3/4 of time +)	41	21	19	35
Working to tight deadlines (3/4 of time +)	37	30	25	35
Three or more pace determinants	34	30	20	28
**Time pressure (Mean)**	37.33	27	21.33	32.67
Hide emotions (most of time/always)	36	37	35	44
Handling angry clients (3/4 of time +)	21	19	23	28
Emotionally disturbing situations (3/4 of time +)	9	14	13	24
**Task complexity (Mean)**	22	23.33	23.67	32.00

Source, Sixth European Working Conditions Survey (Eurofound, 2016); Index from 0 to 100%.

Likewise, Karasek (1979) pointed out the ‘tendency to describe all structurally determined work characteristics as job demands regardless of their drastically different effects on psychological functioning’ and also that a more specific distinction between job demands is needed due to the ‘inconsistent finding that time pressure demands are associated with strain symptoms, while intellectual demands are not’ (p. 286). This consideration suggests differences between effects on employees’ health of intellectual-cognitive job demands and other job demands such as time pressure based first on the aforementioned premise pointed out by Karasek (1979) about differences between the effects of job demands on employees’ health. Second, Bakker and Demerouti (2013) consideration that job demands involve a sustained effort with a physical and psychological toll. And third, effects of job characteristics on employees’ health and distinction between psychological and physical symptoms of Väänänen et al. (2003).

Previous findings indicate that each job activity, and its specific job characteristics has specific psychosocial health determinants (Väänänen et al., 2003). Within the service sector, there are occupations that could differ in the type of demands they receive from users, in physical overload, and to what extent work interferes with employee personal life (Eurofound and EU-OSHA, 2014). Similarly, employees in these occupations differ in their level of education and tenure and the perceived stability of their contract (Eurofound, 2016). All these aspects could influence the link between job demands and employees’ health. In this sense, the employees of the education and healthcare subsectors generally receive high emotional demands from users (Szczygieł and Bazińska, 2013). These occupations usually spillover to personal life because there are less chances of recovering from work activity (Lockwood, 2003).

Similarly, job occupations could also differ in the level of education of employees, with a higher level of employee education in the public administration, education, and healthcare subsectors (Eurofound, 2016). Educational level could modify the personal appraisal of job demands, considering moderated levels of job demands as a challenge (LePine et al., 2005; Van den Broeck et al., 2010). In other job occupations with more unstable employment and lower educational level, job demands could be perceived by employees as a hindrance (LePine et al., 2005; Van den Broeck et al., 2010). Additionally, the JD-R Model (Bakker and Demerouti, 2007) predicts that job resources, as job autonomy, protect employees’ health against the negative influence of job demands. Likewise, job autonomy was defined by Hackman and Oldham (1975) as ‘the degree to which the job provides substantial freedom, independence, and discretion to the employee in scheduling the work and in determining the procedures to be used in carrying it out’ (p. 162).

Based on these premises, we could expect differences depending on the service subsectors analyzed. Thus in education and healthcare subsectors due to a more stable employment consequence of stable contracts as public servants in large public organizations (as public hospitals, high schools, and universities), higher requirements of educational level and higher level of job autonomy, the job demands related to the task, such as task complexity, could produce a lower negative influence on health when job demand levels are higher. Consequently, we could predict lower and linear effects of job demand on employees’ health in the education and healthcare subsectors However, in the commerce and horeca subsectors where most of the Spanish companies in these subsectors are private small companies, offering unstable employment and low pay rates, with low requirements of educational level and low level of job autonomy, we could predict higher and non-linear effects of job demand on employees’ health. Similarly, we could predict higher and non-linear effects of job demand on employees’ health in public administration due to low level of job autonomy as a specific subsector characteristic with a highly hierarchical and inflexible bureaucratic work system. Regarding the shape of non-linear relationships, as psychological and physical symptoms are negatively related to health and wellbeing, thus the higher the symptoms the lower the health, we could predict a U-shape. Because there is no previous evidence regarding the effect of job demands using a broad occupational analysis, we will elaborate our hypotheses with an exploratory character. Thus, regarding these precedents, we propose the following hypotheses:

**Hypothesis 1:** The linear and non-linear relationships between specific job demands and psychological and physical symptoms of employees will differ depending on the service subsectors analyzed.

**Hypothesis 2:** When the effect of job demands-resources on psychological and physical symptoms of employees is non-linear a U-shaped relationship is expected.

## Materials and methods

### Sample and procedures

To carry out this study, a systematic review of recent literature related to non-linear relationships between job demands-resources and their outcomes was carried out first. In order to systematize our literature exploration and analyses, we followed the recommendations of the Cochrane Handbook for Systematic Reviews (Higgins and Green, 2008). To answer the question ‘which type of designs and methodologies are used in recent studies on non-linear relationships between job demands and their outcomes?’, we applied a specification of criteria for including and excluding studies in the literature review. The eligibility criteria were as follows: journals indexed in Web of Science (WoS) or SCOPUS, with high quality index Q1 or Q2 containing ‘non-linear relationship,’ ‘curvilinear relationship,’ and ‘job demands’ as keywords in the article title or in the abstract. Result records identified through database searching were 49 documents found in SCOPUS and 38 documents found in WoS. After removing duplicate articles and evaluating their eligibility, we selected 15 studies (shown in Table 1) to be included in our qualitative synthesis as a significant sample of research in this field.

Data were extracted from the second Andalusian Survey of Working Conditions (II ASWC). The II ASWC was carried out in 2012 on a sample of 8,854 employees. This survey is carried out by the Andalusian Institute for Prevention of Occupational Risks (IAPRL) related to the Andalusia Regional Government, to know the perception that Andalusian employees have about their health in relation to the work activity they perform and the conditions in which they do it, as well as their knowledge about the organization and other preventive activities carried out by companies on their workplaces. The II ASWC survey was directed at the employed population, through a stratified random sampling proportional to the size of the population under study and representative of the employed population resident in municipalities larger than 5.000 inhabitants with a confidence level of 95.5%. The details of the II ASWC are available in a public document edited by the Andalusian Government (Instituto Andaluz de Prevención de Riesgos Laborales [IAPRL], 2012). We have preferred to use a regional survey more than a national or European one, to increase homogeneity in participants and reduce cultural differences. Although a more recent ASWC is not available yet and even some variables could be sensitive to the economic and social context (Sanclemente et al., 2020), their nature is the same and the relationships between them (lineal or non-linear) might be maintained. Furthermore, the II ASWC sample shows a great interest for our study because the Andalusian economy depends mostly on the service sector (Instituto Nacional de Estadística [INE], 2019).

Thus, the final sample used for our study was composed of service sector employees, extracted from the II ASWC of 2012 (*N* = 4047), with a proportion of 52.5% women and 47.5% men, a mean age of 38.01 years (*SD* = 10.41), and a mean job tenure of 8.41 years (*SD* = 9.12). The age ranged from 18 to 65 years, and the job tenure from 6 months to 48 years. Additionally, from this final sample, we extracted five subsamples by service subsectors related to commerce (*n* = 1,498) with a proportion of 50.2% women and 49.8% men, a mean age of 36.18 (*SD* = 10.60) and a mean job tenure of 7.29 (*SD* = 8.47). Horeca (*n* = 655) with a proportion of 46% women and 54% men, a mean age of 34.71 (*SD* = 10.11) and a mean job tenure of 5.34 (*SD* = 7.06). Public administration (*n* = 778) with a proportion of 42.4% women and 57.6% men, a mean age of 41.83 (*SD* = 8.87) and a mean job tenure of 12.1 (*SD* = 10.27). Education (*n* = 519) with a proportion of 58.8% women and 41.2% men, a mean age of 40.08 (*SD* = 9.81) and a mean job tenure of 9.52 (*SD* = 9.28). And healthcare (*n* = 597) with a proportion of 72.9% women and 27.1% men, a mean age of 39.45 (*SD* = 10.5) and a mean job tenure of 8.85 (*SD* = 9.19). It should be noted that in the healthcare and education subsectors, the proportion of women is higher than that of men, whereas the opposite occurs in public administration. Regarding job tenure, employees in the horeca subsector showed the lowest mean job tenure, suggesting that this sector is not as stable as the rest of subsectors analyzed.

### Measures

In terms of job demands, two general job demands were selected, task complexity and time pressure, and a specific job demand found in the service sector, which is contact with users. Likewise, job autonomy was considered as a job resource. Scales and items used to measure task complexity, time pressure, and contact with users were extracted from the Spanish version of the Copenhagen Psychosocial Questionnaire (COPSOQ) (Moncada et al., 2005). In addition, the employees also reported the number of symptoms they suffered recently and most frequently. In order to measure the psychosomatic symptoms of the employees, we distinguished between psychological symptoms and physical symptoms. Based on Väänänen et al. (2003), our criteria for classifying the symptoms into both categories were made based on whether the symptoms reported by employees were manifested on a physical level (soma), i.e., perception of discomfort, pain, or fatigue in a particular part of the employee’s physical body, or whether the symptom was manifested as the perception of some kind of discomfort on a psychological or mental level (psyche), i.e., low mood or feeling tense and irritable. Although some physical symptoms considered in our study could be of mental or emotional origin, they are experienced by employees as pain or physical discomfort (Ford et al., 2011). However, determining the origin of these symptoms was not part of the objective of this study. As it is well established in cross-sectional studies psychological and physical symptoms predict employee absenteeism due to sickness (Väänänen et al., 2003). Nevertheless, longitudinal study conducted by Väänänen et al. (2003) suggests that symptoms first cause short absences, and in long-term (9 months to 1 year) sick leaves prescribed by a physician. Thus, the relationship between psychosomatic symptoms and employees’ health is not direct and requires a relatively long process influenced by many factors (Väänänen et al., 2003). Finally, the Cronbach alpha values for the scales reached the threshold of (α = 0.70) suggested by Nunally (1978), showing good internal consistency.

#### Task complexity

Following Liu and Li (2012) objective definition of tax complexity as the aggregation of intrinsic task characteristics influencing the performance of a task, task complexity was measured with five items like the following: ‘To what extent does your job involve perform complex, complicated, or difficult tasks?’ The response ranged from 1 ‘Almost never or never’ to 5 ‘Always or almost always.’ The five items of task complexity regarding intrinsic task characteristics were related to sensory, cognitive, and emotional psychological demands as requirements of high level of attention, performing complicated or difficult tasks, working with computers, intellectually demanding jobs, and emotionally demanding jobs. The factor structure was tested twice through exploratory factor analysis (EFA) and confirmatory factor analysis (CFA). EFA showed a one-factor solution and a Kaiser–Meyer–Olkin (KMO) index of 0.72 above values considered by Kaiser (1974) as satisfactory for factor analysis (between 0.70 and 0.80). The Bartlett sphericity test was significant (χ^2^ = 4011.39, *df* = 10, *p* < 0.001) showing data suitability for factor analysis. The one-factor CFA showed an adequate fit to the data (χ^2^ = 201.11, *df* = 5, *p* < 0.001, RMSEA = 0.088, CFI = 0.96, GFI = 0.98, NFI = 0.96). Additionally, factor loadings ranged from 0.49 to 0.83 above the minimum factor loading cut-off of 0.30 commonly accepted in factor analysis (Bandalos and Finney, 2010). Cronbach alpha coefficient was 0.71.

#### Time pressure

It was measured with four items like the following: ‘To what extent does your job involve working very fast?’ The response ranged from 1 ‘Almost never or never’ to 5 ‘Always or almost always.’ The four items of time pressure were related to working fast, working with tight deadlines, doing repetitive tasks in a very short time, and performing various tasks at the same time. EFA showed a one factor solution, a KMO index of 0.75, and a significant Bartlett sphericity test (χ^2^ = 3356.01, *df* = 6, *p* < 0.001) showing data suitability for factor analysis. The one-factor CFA showed an adequate fit to the data (χ^2^ = 24.08, *df* = 2, *p* < 0.001, RMSEA = 0.052, CFI = 0.99, GFI = 0.99, NFI = 0.99). Additionally, factor loadings were adequate, ranging from 0.68 to 0.79. Cronbach’s alpha coefficient was 0.74.

#### Contact with users

It was measured with a single item: ‘To what extent does your job involve dealing directly with people who are not employees?’ The response ranged from 1 ‘Almost never or never’ to 5 ‘Always or almost always.’ Contact with users is related to contact with clients, users, or pupils. Considering that the concept is simple and clear, the use of a single item to measure it was well justified (Fisher et al., 2016).

#### Job autonomy

It was measured with four items like the following: ‘At your job, can you choose or change the order of tasks?’ The response ranged from 1 ‘Almost never or never’ to 5 ‘Always or almost always.’ The four items of job autonomy were related to the possibility of change or modify the order of tasks, the rhythm of work, the distribution, and/or duration of work breaks, and put your own ideas into practice at work. EFA showed a one-factor solution, a KMO index of 0.77, and a significant Bartlett sphericity test (χ^2^ = 6810.03, *df* = 6, *p* < 0.001) showing data suitability for factor analysis. The one-factor CFA showed an adequate fit to the data (χ^2^ = 72.51, *df* = 2, *p* < 0.001, RMSEA = 0.093, CFI = 0.99, GFI = 0.99, NFI = 0.99). Furthermore, the factor loadings were adequate ranging from 0.59 to 0.88. Cronbach’s alpha coefficient was 0.82.

#### Psychosomatic symptoms

It was measured by showing to the participants a list of eleven symptoms and then asking them to report the number of symptoms on the list that they had suffered recently and frequently. Subsequently, symptoms were classified as five types of psychological symptoms and six types of physical symptoms, generating two separated variables as follows. *Psychological symptoms:* the five types of psychological symptoms considered were: sleep problems, difficulty concentrating, forgetting things, feeling tense/irritable, low mood. Each case could present a minimum of zero to a maximum of five psychological symptoms. Therefore, this quantitative variable represents the number of psychological symptoms reported by each employee. *Physical symptoms:* the six types of physical symptoms considered were: feeling tired, having headaches, dizziness, digestive disorders, eye problems, or other muscle aches. Each case could present a minimum of zero to a maximum of six physical symptoms. Therefore, this quantitative variable represents the number of physical symptoms reported by each employee. Considering each symptom on the initial list as a dichotomic item (presence or absence of symptom), reliability was assessed applying the Kuder–Richardson 20 (KR20) reliability test for dichotomic items (Kuder and Richardson, 1937). Reference values for this index are the same as the Cronbach’s alpha coefficient. The Kuder–Richardson index (KR20) was 0.89 showing adequate reliability. ANOVA applying Cochran’s *Q* test for dichotomic items was significant [*Q*_(9,4046)_ = 11520.60, *p* < 0.001] showing statistically significant between-subjects discrimination.

#### Control variables

Occupational gender roles and age show a significant relation to workplace stress and mental health in workers (Kerr et al., 2021). Furthermore, job tenure is associated with symptoms of musculoskeletal disorder (Warren et al., 2015). Thus, gender, age, and job tenure were selected as control variables due to their relationship with the psychological and physical symptoms of our study output variables. Before including the gender variable in our study, it was converted into a dummy variable that assigns a cero value to men and one value to women.

### Data analysis

In order to test our hypotheses about non-linear relationships, we conducted hierarchical multiple regression analysis (Cohen et al., 2003). Our study variables were introduced in three successive steps. In the first step, control variables such as gender, age and job tenure; in a second step, linear variables (task complexity, time pressure, and contact with users); and finally, in a third step, quadratic variables (task complexity squared, time pressure squared, and contact with users squared). Variables were mean-centered before we calculated multiple regression models (Cohen et al., 2003). Models with a significant incremental of *R*^2^ (Δ*R*^2^), when squared variables are entered in the model, signal a significant incremental variance adding explanatory power to the model (Cohen et al., 2003). Effect size was reported as Cohen’s *d* statistic (Cohen, 1988).

## Results

Table 3 presents means, standard deviations, and correlations for the study variables. Regarding the correlations for the job demand variables first, task complexity and time pressure were significant and positively related to physical symptoms and psychological symptoms. Second, contact with users was significant and positively related to psychological symptoms. Regarding the correlations for job resources, job autonomy was significant and positively related to task complexity and contact with users, and negatively related to time pressure. Furthermore, both physical and psychological symptoms were significant and positively related.

**TABLE 3:** Descriptive statistics and correlations for the study variables.

Variable	Mean	*SD*	1	2	3	4	5	6	7	8	9
(1) Gender	−	−	−								
(2) Age	38.01	10.41	−0.11**	−							
(3) Job tenure	8.41	9.12	−0.12**	0.64**	−						
(4) Task complexity	3.18	0.94	0.02	0.04**	0.13**	−					
(5) Time pressure	3.05	0.98	0.00	−0.08**	–0.01	0.42**	−				
(6) Contact with users	4.36	1.14	0.07**	−0.08**	−0.07**	0.14**	0.03	−			
(7) Job autonomy	3.38	1.10	–0.02	0.15**	0.09**	0.10**	−0.05**	0.10**	−		
(8) Physical symptoms	0.64	0.94	0.11**	0.06**	0.06**	0.14**	0.16**	0.02	0.01	−	
(9) Psychological symptoms	0.39	0.85	0.06**	0.08**	0.06**	0.14**	0.10**	0.04**	0.02	0.50**	–

**p < 0.01; two tailed; (N = 4047).

Finally, regarding the correlations for control variables, gender, age, and job tenure were significant and positively related to physical and psychological symptoms. Additionally, a mean comparison analysis showed some gender differences. Therefore, women in the service sector (*N* = 4047) showed a significant higher number of physical and psychological symptoms than men [physical symptoms: women: *M* = 0.74, *SD* = 1.03; men: *M* = 0.53, *SD* = 0.84; *t* = 7.12, *p* < 0.001; effect size: *d* = 0.22); psychological symptoms: women: *M* = 0.45, *SD* = 0.92; men: *M* = 0.34, *SD* = 0.76; *t* = 4.09, *p* < 0.001; effect size: *d* = 0.13)].

### Relationships between job demands-resources and psychological and physical symptoms by sectors

For commerce (Table 4), regarding psychological symptoms, the results of Model 5 showed positive linear relationships among task complexity, time pressure, contact with users, and psychological symptoms. Likewise, Model 5 showed a positive non-linear relationship between task complexity and psychological symptoms. Regarding physical symptoms, the results of Model 5 showed a positive linear relationship between time pressure and physical symptoms.

**TABLE 4:** Hierarchical multiple regression analysis models predicting psychological and physical symptoms in commerce.

	Psychological symptoms						Physical symptoms					
Variables	M1	M2	M3	M4	M5	*d*	M1	M2	M3	M4	M5	*d*
Gender	0.13**	0.12**	0.13**	0.12**	0.12**	0.24	0.11**	0.11**	0.11**	0.11**	0.11**	0.22
Age	0.10**	0.11**	0.09**	0.09**	0.09**	0.14	0.11**	0.12**	0.11**	0.11**	0.11**	0.17
Job tenure	–0.02	–0.02	–0.03	–0.02	−0.02		–0.02	–0.02	–0.02	–0.02	−0.02	
Task complexity		0.07*	0.06*	0.08*	0.08**	0.14		0.02	0.02	0.02	0.02	
Time pressure		0.08**	0.09**	0.09**	0.09**	0.16		0.14**	0.15**	0.15**	0.15**	0.26
Contact with users		0.05*	0.04	0.13*	0.12*	0.11		0.03	0.02	0.10	0.10	
Job autonomy			0.09**	0.07**	0.08**	0.14			0.05*	0.05	0.05	
Task complexity Sq				0.06**	0.06*	0.12				0.01	0.01	
Time pressure Sq				0.02	0.02					0.01	0.01	
Contact with users Sq				0.08	0.08					0.08	0.08	
Job autonomy Sq					0.02						0.01	
*R* ^2^	0.022	0.037	0.048	0.055	0.055		0.018	0.042	0.045	0.046	0.046	
Δ*R*^2^	0.022**	0.019**	0.007**	0.007*	0.000		0.018**	0.024**	0.002*	0.001	0.000	

*p < 0.05, **p < 0.01; β standardized regression coefficients are displayed; M, model; Sq, squared; d, effect size for M5 coefficients; (n = 1498).

For horeca (Table 5), regarding psychological symptoms, Model 5 results showed positive linear relationships among task complexity, contact with users and psychological symptoms. Additionally, Model 5 showed positive non-linear relationships among task complexity, contact with users and psychological symptoms. In reference to physical symptoms, Model 5 results showed positive linear relationships among task complexity, time pressure, and physical symptoms. Likewise, Model 5 showed a positive non-linear relationship between task complexity and physical symptoms.

**TABLE 5:** Hierarchical multiple regression analysis models predicting psychological and physical symptoms in horeca.

	Psychological symptoms						Physical symptoms					
Variables	M1	M2	M3	M4	M5	*d*	M1	M2	M3	M4	M5	*d*
Gender	0.04	0.05	0.05	0.06	0.06		0.08*	0.09*	0.09*	0.09*	0.09*	0.19
Age	0.07	0.08	0.08	0.09*	0.09*	0.17	0.07	0.09*	0.10*	0.10*	0.10*	0.18
Job tenure	–0.01	–0.03	–0.03	–0.04	−0.04		0.07	0.07	0.07	0.07	0.07	
Task complexity		0.21**	0.21**	0.22**	0.23**	0.43		0.13**	0.14**	0.14**	0.15**	0.27
Time pressure		–0.01	–0.01	–0.03	−0.04			0.15**	0.14**	0.13**	0.13**	0.23
Contact with users		–0.01	–0.01	0.18*	0.18*	0.16		0.01	0.01	–0.01	−0.01	
Job autonomy			–0.01	–0.03	−0.04				–0.06	–0.07	−0.07	
Task complexity Sq				0.18**	0.18**	0.36				0.08*	0.08*	0.16
Time pressure Sq				–0.04	−0.04					–0.04	−0.05	
Contact with users Sq				0.22*	0.21*	0.19				–0.02	−0.02	
Job autonomy Sq					0.05						0.03	
*R* ^2^	0.006	0.050	0.050	0.093	0.095		0.019	0.074	0.077	0.085	0.085	
Δ*R*^2^	0.006	0.044**	0.000	0.043**	0.002		0.019**	0.055**	0.003	0.007	0.001	

*p < 0.05, **p < 0.01; β standardized regression coefficients are displayed; M, model; Sq, squared; d, effect size for M5 coefficients; (n = 655).

For public administration (Table 6), in terms of psychological symptoms, the results of Model 5 showed a positive linear relationship between task complexity and psychological symptoms and a negative linear relationship between job autonomy and psychological symptoms. Additionally, Model 5 showed a positive non-linear relationship between task complexity and psychological symptoms. Regarding physical symptoms, Model 5 results showed positive linear relationships between task complexity, time pressure, and physical symptoms, and a negative linear relationship between job autonomy and physical symptoms. Likewise, Model 5 showed a positive non-linear relationship between task complexity and physical symptoms.

**TABLE 6:** Hierarchical multiple regression analysis models predicting psychological and physical symptoms in public administration.

	Psychological symptoms						Physical symptoms					
Variables	M1	M2	M3	M4	M5	*d*	M1	M2	M3	M4	M5	*d*
Gender	0.04	0.03	0.03	0.04	0.04		0.12**	0.11**	0.11**	0.12**	0.12**	0.24
Age	0.01	0.05	0.07	0.04	0.04		–0.01	0.03	0.04	0.02	0.02	
Job tenure	0.09*	0.04	0.03	0.05	0.05		0.13*	0.08	0.07	0.08	0.08	
Task complexity		0.14**	0.14**	0.21**	0.21**	0.31		0.12**	0.12**	0.18**	0.18**	0.26
Time pressure		0.07	0.08	0.07	0.07			0.09*	0.09*	0.09*	0.09*	0.15
Contact with users		0.01	0.01	0.06	0.05			–0.06	–0.05	–0.01	−0.01	
Job autonomy			−0.09*	−0.11**	−0.11**	−0.21			−0.07*	−0.08*	−0.08*	−0.17
Task complexity Sq				0.13**	0.13**	0.21				0.09*	0.09*	0.15
Time pressure Sq				0.01	0.01					–0.01	0.00	
Contact with users Sq				0.05	0.05					0.06	0.06	
Job autonomy Sq					0.01						−0.01	
*R* ^2^	0.011	0.045	0.053	0.068	0.068		0.025	0.055	0.060	0.069	0.069	
Δ*R*^2^	0.011*	0.034*	0.008*	0.015**	0.000		0.025**	0.031**	0.005*	0.009	0.000	

*p < 0.05, **p < 0.01; β standardized regression coefficients are displayed; M, model; Sq, squared; d, effect size for M5 coefficients; (n = 778).

For education (Table 7), related to psychological symptoms, the results of Model 5 showed a positive linear relationship between task complexity and psychological symptoms. Similarly, Model 5 showed a positive non-linear relationship between time pressure and psychological symptoms. Regarding physical symptoms, the results of Model 5 showed a positive linear relationship between task complexity and physical symptoms.

**TABLE 7:** Hierarchical multiple regression analysis models predicting psychological and physical symptoms in education.

	Psychological symptoms						Physical symptoms					
Variables	M1	M2	M3	M4	M5	*d*	M1	M2	M3	M4	M5	*d*
Gender	–0.01	0.02	0.02	0.02	0.02		0.17**	0.19**	0.19**	0.19**	0.19**	0.39
Age	0.14*	0.19**	0.19**	0.20**	0.20**	0.31	0.08	0.12*	0.12*	0.12*	0.12*	0.19
Job tenure	–0.01	–0.07	–0.07	–0.07	−0.06		0.02	–0.03	–0.03	–0.03	−0.03	
Task complexity		0.23**	0.24**	0.26**	0.26**	0.43		0.19**	0.19**	0.22**	0.22**	0.35
Time pressure		–0.01	–0.01	–0.03	−0.03			0.01	0.01	0.00	0.00	
Contact with users		–0.01	–0.01	0.09	0.09			–0.01	–0.01	0.05	0.05	
Job autonomy			–0.06	–0.07	−0.07				0.01	0.01	0.01	
Task complexity Sq				0.03	0.03					0.04	0.04	
Time pressure Sq				0.12**	0.12**	0.25				0.00	0.00	
Contact with users Sq				0.12	0.11					0.07	0.07	
Job autonomy Sq					0.06						−0.01	
*R* ^2^	0.018	0.065	0.068	0.090	0.093		0.036	0.072	0.072	0.076	0.076	
Δ*R*^2^	0.018*	0.047**	0.003	0.021**	0.003		0.036**	0.036**	0.000	0.003	0.000	

*p < 0.05, **p < 0.01; β standardized regression coefficients are displayed; M, model; Sq, squared; d, effect size for M5 coefficients; (n = 519).

Finally, in healthcare (Table 8), regarding psychological symptoms, the results of Model 5 showed a positive linear relationship between time pressure and psychological symptoms. Likewise, Model 5 showed a positive non-linear relationship between job autonomy and psychological symptoms. Regarding physical symptoms, Model 5 results showed positive linear relationships among task complexity, time pressure, and physical symptoms. Furthermore, Model 5 showed a positive non-linear relationship between job autonomy and physical symptoms.

**TABLE 8:** Hierarchical multiple regression analysis models predicting psychological and physical symptoms in healthcare.

	Psychological symptoms						Physical symptoms					
Variables	M1	M2	M3	M4	M5	*d*	M1	M2	M3	M4	M5	*d*
Gender	0.09*	0.08*	0.08*	0.08*	0.08*	0.17	0.16**	0.15**	0.15**	0.15**	0.16**	0.32
Age	0.01	0.04	0.05	0.04	0.04		–0.08	–0.03	–0.03	–0.04	−0.05	
Job tenure	0.18**	0.15**	0.14*	0.14*	0.14**	0.22	0.17**	0.11*	0.11*	0.11*	0.11*	0.18
Task complexity		0.07	0.07	0.09	0.09			0.14**	0.0.14**	0.15**	0.15**	0.25
Time pressure		0.09*	0.09*	0.10*	0.10*	0.19		0.16**	0.16**	0.16**	0.16**	0.30
Contact with users		–0.01	–0.01	0.06	0.04			–0.05	–0.05	–0.14	−0.15	
Job autonomy			–0.06	–0.06	−0.04				0.01	0.01	0.03	
Task complexity Sq				0.06	0.05					0.05	0.04	
Time pressure Sq				–0.01	-0.02					–0.02	−0.02	
Contact with users Sq				0.06	0.05					–0.11	−0.11	
Job autonomy Sq					0.09*	0.18					0.09*	0.18
*R* ^2^	0.039	0.057	0.061	0.064	0.071		0.042	0.099	0.099	0.102	0.110	
Δ*R*^2^	0.039**	0.018*	0.003	0.003	0.007*		0.042**	0.057**	0.000	0.003	0.008*	

*p < 0.05, **p < 0.01; β standardized regression coefficients are displayed; M, model; Sq, squared; d, effect size for M5 coefficients; (n = 597).

Linear and non-linear relationships between job demands-resources and psychological and physical symptoms, and the total variance (*R*^2^) explained by each model in the five service subsectors analyzed evidenced differences among the five subsectors. Moreover, significant relationships among job demands-resources and physical and psychological symptoms were different in each subsector, mixed linear and non-linear, as shown in Tables 4–8.

**FIGURE 1:**
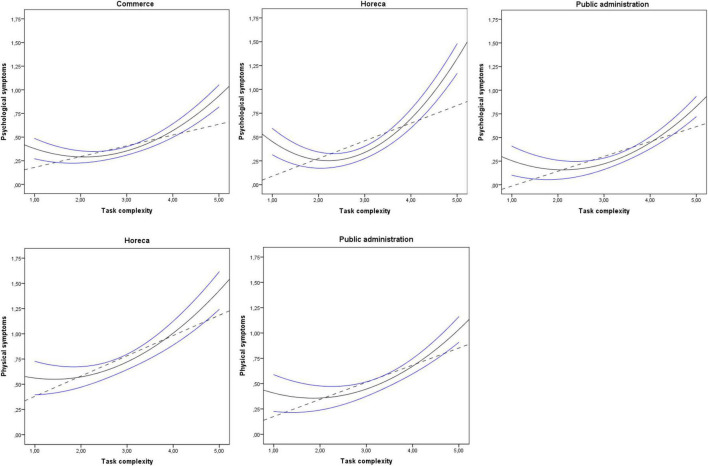
Linear and non-linear significant effects on physical and psychological symptoms of task complexity in commerce, horeca and public administration. Discontinuous line shows linear relationship. Continuous line shows squared relationship. Blue lines above and below continuous black curve line signal 95% CI upper bound and 95% CI lower bound, respectively.

**FIGURE 2:**
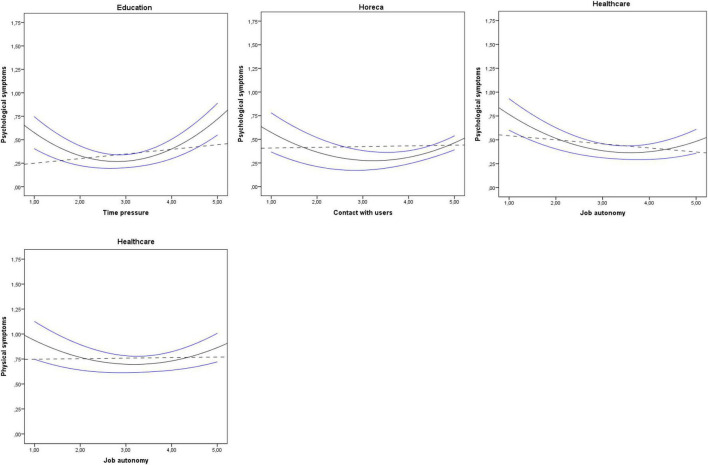
Linear and non-linear significant effects of time pressure in education, contact with users in horeca and job autonomy in healthcare. Discontinuous line shows linear relationship. Continuous line shows squared relationship. Blue lines above and below continuous black curve line signal 95% CI upper bound and 95% CI lower bound, respectively.

Finally, graphics showing linear and non-linear relationships among task complexity, time pressure, contact with users, and psychological and physical symptoms are represented in Figures 1, 2. The results shown in Figure 1 revealed curvilinear relationships between task complexity and psychological symptoms in commerce, horeca, and public administration, and between task complexity and physical symptoms in horeca and public administration. Additionally, the results shown in Figure 2 indicated curvilinear relationships between time pressure and psychological symptoms in education, between contact with users and psychological symptoms in horeca, and between job autonomy and psychological and physical symptoms in healthcare. The non-linear relationships found followed a U-shape or a J-shape with a significant incremental of *R*^2^ for the squared variables entering the model.

Regarding H1 about a service subsector and their specific activities affecting the linear or non-linear relationship between specific job demands and psychological and physical symptoms, our findings suggest that the same job demand-resource does not act equally in the different service subsectors in relation to their linear or non-linear effects. For instance, task complexity showed non-linear effects on psychological symptoms in commerce, horeca, and public administration. However, the effects of task complexity were linear in education and not significant in healthcare. With respect to H2, as expected, non-linear U-shaped relationships were found. Therefore, the U-shaped relationships found indicate that low and moderate levels of job demand produced the lowest negative impact on psychological and physical symptoms. Furthermore, a curvilinear model predicts relationships between job characteristics and their outcomes better than a linear model. Thus, squared variables introduced produce a significant incremental of *R*^2^ (Δ*R*^2^) adding explanatory power to the model.

## Discussion

Our findings suggest that linear and non-linear relationships between specific job demands-resources, and psychological and physical symptoms of employees were different depending on the service subsectors analyzed. Likewise, task complexity showed non-linear relationships to psychological and physical symptoms of employees in commerce, horeca and public administration. However, time pressure and contact with users only produced non-linear effects in education and horeca, respectively. Furthermore, job autonomy showed a protective pattern of health, linear in public administration and non-linear in healthcare. In addition, as the Vitamin Model (Warr, 1987) predicts, when the effects of task complexity, time pressure, and contact with users on psychological and physical symptoms were curvilinear they followed an AD pattern, through a cumulative effect leading into impaired health (like vitamins A and D), increasing psychological and physical symptoms, and following a U-shape in our case. The results agree with previous literature that analyzes psychosocial health predictors considering the influence of employment sectors and occupations (Väänänen et al., 2003). Regarding a possible explanation of differences among activity sectors in linear and non-linear relationships between job demands-resources and organizational outcomes, Bakker and Demerouti (2013) suggested the existence of general job demands, which are common to most jobs, and specific job demands in each activity sector. Likewise, job demands such as work pressure or emotionally demanding relationships with clients are not always necessarily negative, and they become an obstacle when they require a great effort on the part of the worker without being sufficiently recovered resulting in an exhaustion process (Bakker and Demerouti, 2013). In this line, the approach to work stressors from the challenge-hindrance perspective, considering the negative and positive aspects of job demands simultaneously, indicates that challenging job demands have positive effects on employee wellbeing and motivation despite their potential as stressors, on the contrary, hindrance job demands reduce employee wellbeing and motivation (LePine et al., 2005). Considering these approaches to job demands-resources it might be possible that specific service sector job demands, which were considered challenging by employees such as moderate levels of contact with users, time pressure, and task complexity, would show fewer negative impact on their psychological symptoms. On the other hand, it might be possible that when these job demands were considered as a hindrance by employees, they had a greater negative impact on their psychological symptoms, as may occur when employees are exposed to high levels of contact with users, time pressure, and task complexity. Furthermore, our findings were also compatible with previous research from the challenge-hindrance perspective on the effects of time pressure on employee outcomes (Schmitt et al., 2015), and with studies combining the approach from the JD-R model and from the challenge-hindrance perspective and their positive and negative effects on employee wellbeing and health (Van den Broeck et al., 2010; Tadić et al., 2015).

The findings also suggest that older employees reported a greater impact of job demands on psychological and physical symptoms than younger ones in some sectors such as education and commerce. According to the literature, this could be a consequence of burnout levels in the case of education or physical difficulties in developing some tasks due to age in commerce (Aittomäki et al., 2005). Furthermore, we found a negative effect of job tenure on employees’ health in the healthcare subsector. According to the literature, this could also be due to the burnout level in doctors and nurses (Aittomäki et al., 2005). Finally, some gender differences were found. Women showed a significantly higher number of physical and psychological symptoms than men. These gender differences may be due to the fact that women are socially forced to adapt work to family demands in order to achieve a work-life balance (Burke and McKeen, 1994; Lockwood, 2003; Kelliher et al., 2019). In this sense, in Europe, 70% of the workers in education and healthcare sectors are women (Eurofound and EU-OSHA, 2014). A good work-life balance, for both men and women, appears as a solution to this problem and helps decreasing the chances of reporting health problems (Burke and McKeen, 1994; Lockwood, 2003; Eurofound and EU-OSHA, 2014; Kelliher et al., 2019).

### Practical implications

Based on our results, we propose here some general recommendations for human resources managers (HRM) and practitioners on employees’ exposure levels to the three job demands studied. Our recommendations aim to reduce the negative impact of job demands on the psychosomatic symptoms of service sector employees and their associated absenteeism (Väänänen et al., 2003). According to this study, the level of job demands that can be disruptive and generate negative effects on psychological and physical symptoms differs on the sector of activity. In this sense, considering relationships between job demands-resources and psychological and physical symptoms found, we recommend to HRM that job demands to which employees are exposed should not exceed medium levels in order to reach the lowest negative impact on psychological and physical symptoms, preserving their health. This is based on when relationships are non-linear an exponential increase in symptoms occurs if the midpoint is crossed, as our findings signal. Consequently, HRM should avoid maintaining high or extremely high levels of task complexity and time pressure for a long period of time to reduce the psychological and physical symptoms of employees, preventing absenteeism and possible future negative effects on employees’ health (Väänänen et al., 2003; Žutautienė et al., 2020). Furthermore, HRM should pay attention to the level of job autonomy of employees in public administration and healthcare, in order to buffer the negative effects of job demands on employees’ health.

Some examples of practical suggestions related to regulation of job demand levels are related to intrinsic characteristics of job tasks. In this sense, job analysis is a necessary tool for managers to increase health levels. Regulation of task complexity can be done by changing job characteristics such as the necessary level of attention, intrinsic task difficulty, and emotional and intellectual requirements. In the case of jobs with computers, software applications, and tasks, these guidelines should be followed as well. Regulation of time pressure levels can be done by changing job characteristics such as required work performance speed, deadlines, repetitive tasks, and number of tasks done at the same time. And finally, in order to regulate the level of contact with users, we suggest achieving an adequate ratio of employees in relation to the quantity of customers-users-clients-pupils to be attended.

Based on theories reviewed in this paper and regarding to perception of job tasks, the challenge-hindrance perspective signals that a challenging job demand could produce a positive effect on employees’ wellbeing, motivation, and health (LePine et al., 2005; Van den Broeck et al., 2010; Tadić et al., 2015). Introducing job design practices or specific training programs for employees to enhance the intrinsic characteristics of their job could improve the competences and motivation of employees. An adequate job description could be also useful for this purpose. This document is usually handed to employees at the beginning and contains essential job requirements and demands, job duties, job responsibilities, and skills required to perform a specific role (Pató, 2015). As a result, these practices could change employees’ perceptions of a specific level of task complexity, time pressure, and contact with users from a hindrance to a challenge with positive effects for employees and organizations. Additionally, tools as job description could be used for job recruitment in order to find employees with appropriate competences and values in order to deal with job demands and organizational expectations and values (Pató, 2017). Similarly, regarding employees’ resources and the JD-R Model (Bakker and Demerouti, 2007), increasing employees’ job autonomy HRM and practitioners could reduce the negative impact of job demands on employees. The analysis of how personal and social resources could modulate the incidence of relationships between job demands and health is a challenge. Future studies should examine this question considering a wide range of job demands-resources. Likewise, the levels of job demand proposed here should be adapted by HRM and practitioners to their specific areas of interest considering all factors involved, as the intrinsic characteristics of each employee, the job tasks, and the job context. Additionally, organizational assessment of job demands-resources levels following the JD-R Model (Bakker and Demerouti, 2007) should consider non-linear effects, incorporating during the risk assessment process non-linear effects of job demand on health of employees (Karanika-Murray et al., 2009). Thus, occupational risk-prevention plans elaborated and applied by HRM should consider non-linear effects during the risk assessment process.

To sum up, our practical recommendations include first, job demands to which employees are exposed should not exceed medium levels, especially when job demands-health relationships are non-linear. Second, if medium levels are exceeded, it is necessary to avoid maintaining high or extremely high levels of task complexity and time pressure for a long period of time. Third, increase job autonomy of employees and its protective effect especially in public administration and healthcare subsectors. Fourth, introducing job design practices and specific training programs for employees to improve their competences and motivation, and to decrease perception of task complexity. Fifth, applying an adequate job description in recruitment and socialization process to achieve proper level of workplace-person adjustment. Finally, occupational risk-prevention plans should consider non-linear effects of job demands on health during the risk assessment process.

### Limitations and future research

This study has some limitations. First, due to the cross-sectional design of our study, we cannot draw conclusions about causal relationships between variables. In this sense, a longitudinal design could help to test our research findings in the future. Second, the data were obtained only through self-report measures with the result of a subjective nature of the data (Podsakoff et al., 2003). Future studies considering objective data would improve this research. Third, possible deviations from normality could cause statistical errors, but the use of large samples (*N* > 80) avoids this problem (Sainani, 2012), as does this study (*N* = 4047). Fourth, results of non-linear relationships between job demands-resources and psychological and physical symptoms of service sector employees and their subsectors are inconclusive about the cause of differences between subsectors in these relationships. Therefore, additional research is needed in this sense from a challenge-hindrance perspective to determine the cause of these differences.

## Conclusion

Considering the limitations mentioned, our main conclusions were as follows. First, our findings indicate differences between subsectors in terms of linear and non-linear relationships among task complexity, time pressure, contact with users, job autonomy, and physical and psychological symptoms of employees. Second, in the service sector, task complexity, time pressure, contact with users, and job autonomy produced significant linear and non-linear relationships (U-shaped or J-shaped) with employees’ psychological and physical symptoms. Furthermore, task complexity generated non-linear relationships in higher proportion than time pressure and contact with users. These differences in the relationships between job demands-resources and psychological and physical symptoms could suggest differences with respect to the linear or non-linear employee response to exposure to job demands and the use of job resources requiring further research. Third, non-linear relationships indicated that medium levels of job demands should not be exceeded in order to reach the lowest negative impact on psychological and physical symptoms for service sector employees preserving their health. Consequently, the classic idea based on linear models to achieve an adequate level of employees’ health and wellbeing should be revised when the job demand relationships are non-linear.

## Data availability statement

Publicly available datasets were analyzed in this study. The dataset of the second Andalusian Survey of Working Conditions (II ASWC) can be obtained from the Instituto Andaluz de Prevención de Riesgos Laborales (IAPRL) (Andalusian Institute for Occupational Health Prevention) on request to iaprl.cefta@juntadeandalucia.es, https://www.juntadeandalucia.es/empleoformacionytrabajoautonomo/webiaprl/.

## Ethics statement

Ethical review and approval was not required for the study on human participants in accordance with the local legislation and institutional requirements. The patients/participants provided their written informed consent to participate in this study.

## Author contributions

FS, NG, AA, and FM: conceptualization, validation, and visualization. FS and NG: methodology and formal analysis. FS: software, investigation, data curation, and writing—original draft preparation. NG, AA, and FM: resources, writing—review and editing, supervision, and project administration. NG and FM: funding acquisition. All authors read and agreed to the published version of the manuscript.
